# Different Founding Effects Underlie Dominant Blue Eyes (DBE) in the Domestic Cat

**DOI:** 10.3390/ani14131845

**Published:** 2024-06-21

**Authors:** Marie Abitbol, Caroline Dufaure de Citres, Gabriela Rudd Garces, Gesine Lühken, Leslie A. Lyons, Vincent Gache

**Affiliations:** 1 Univ Lyon, VetAgro Sup, 69280 Marcy-l’Etoile, France; 2 Institut NeuroMyoGène INMG-PNMG, CNRS UMR5261, INSERM U1315, Faculté de Médecine, Rockefeller, Université Claude Bernard Lyon 1, 69008 Lyon, France; vincent.gache@univ-lyon1.fr; 3 Antagene, 69890 La Tour de Salvagny, France; cdufauredecitres@antagene.com; 4 Generatio GmbH, 69115 Heidelberg, Germany; gabriela.ruddgarces@generatio.com; 5 Institute of Animal Breeding and Genetics, Justus Liebig University Giessen, 35390 Giessen, Germany; gesine.luehken@agrar.uni-giessen.de; 6 Department of Veterinary Medicine and Surgery, College of Veterinary Medicine, University of Missouri, Columbia, MO 65211, USA; lyonsla@missouri.edu

**Keywords:** feline, eye, coat colour, hair, white spotting, deafness, DBE, pigmentation, Waardenburg syndrome

## Abstract

**Simple Summary:**

Aesthetic traits are major components of modern feline breeds. Each breed is defined by a standard specifying morphology; coat length, texture, and colour; and eye colour. Considering the coat and eye colour may help breeders in managing their breeding stocks to optimise their mating, but it also has an impact on the health and well-being of cats. Indeed, some colours and patterns may be associated with deleterious traits. Recently, a new coat and eye pattern has been described in cats. It has been called “dominant blue eyes (DBE)” and includes one or two blue eyes or particolored eyes and minimal white spotting. Different feline breeding lines were developed for DBE, and in some lineages, deafness has been identified as being associated with this trait. The Altai and Topaz breeds were created using DBE founding cats found in Kazakhstan and Russia. The Celestial breed, recognised in France, was created using a DBE outbred male from Kazakhstan and British shorthair and longhair cats. Other breeds, including the Maine Coon, British, Persian, Siberian, Sphynx, and Munchkin cats, introduced DBE. We have previously identified two variants associated with DBE, and here, we report the discovery of a third DBE variant. Finally, we review the presence of the three DBE variants in 14 feline breeding lines.

**Abstract:**

During the last twenty years, minimal white spotting associated with blue eyes was selected by feline breeders to create the Altai, Topaz, and Celestial breeds. Additionally, certain breeders introduced this trait in their lineages of purebred cats. The trait has been called “dominant blue eyes (DBE)” and was confirmed to be autosomal dominant in all lineages. DBE was initially described in outbred cats from Kazakhstan and Russia and in two purebred lineages of British cats from Russia, as well as in Dutch Maine Coon cats, suggesting different founding effects. We have previously identified two variants in the *Paired Box 3 (PAX3)* gene associated with DBE in Maine Coon and Celestial cats; however, the presence of an underlying variant remains undetermined in other DBE breeding lines. Using a genome-wide association study, we identified a single region on chromosome C1 that was associated with DBE in British cats. Within that region, we identified *PAX3* as the strongest candidate gene. Whole-genome sequencing of a DBE cat revealed an RD-114 retrovirus LTR (long terminal repeat) insertion within *PAX3* intron 4 (namely NC_018730.3:g.206975776_206975777insN[433]) known to contain regulatory sequences. Using a panel of 117 DBE cats, we showed that this variant was fully associated with DBE in two British lineages, in Altai cats, and in some other DBE lineages. We propose that this NC_018730.3:g.206975776_206975777insN[433] variant represents the *DBE^ALT^* (*Altai Dominant Blue Eye*) allele in the domestic cat. Finally, we genotyped DBE cats from 14 lineages for the three *PAX3* variants and showed that they were not present in four lineages, confirming genetic heterogeneity of the DBE trait in the domestic cat.

## 1. Introduction

Canine breeds exhibit a broad spectrum of characteristics, including different body sizes, morphologies, aptitudes, and behavioural traits. In contrast, feline breeds are predominantly distinguished by their coat and eye colours, which have been selectively favoured by humans [1,2]. Among coat and eye colours, blue eyes are largely appreciated by feline breeders and owners (loof.asso.fr, tica.org, and cfa.org). In addition to the *colourpoint* allele (*c^s^*), the *white-spotting* (*w^S^*) and *dominant-white* (*W*) alleles have been shown to be associated with the possibility of having one or two blue eyes in cats [3]. Genetic and molecular mechanisms underlying blue eyes associated with these loci have been elucidated in cats [2,3]. A disturbance in (i) the development and migration of melanoblasts from the neural crest to peripheral sites; (ii) the differentiation of melanoblasts into melanocytes; (iii) the survival of melanocytes; and (iv) the synthesis of melanosomes and melanins can produce, among other things, white spots and/or blue eyes in various species [3,4,5,6,7,8,9]. In mammals, various genes have been documented to be involved in melanoblast migration, survival, and proliferation such as the transcription factors *PAX3* (*Paired* Box 3), *SOX10* (*SRY-Box Transcription Factor 10*), and *MITF* (*Melanocyte-Inducing Transcription Factor*); the WNT signalling pathway; *G protein-coupled endothelin receptor B* (*EDNRB*) and its ligand, *endothelin 3* (*EDN3*); and *receptor tyrosine kinase* (*KIT)* and *KIT-ligand* (*KITLG*) [4].

Variants in *PAX3, SOX10, MITF, EDN3, EDNRB,* and *KITLG* genes have been associated with Waardenburg syndrome (WS) in humans, a group of auditory–pigmentary disorders characterised by various features, including blue eyes or heterochromia (one blue or partially blue eye), sensorineural deafness, a white forelock, and the lateral displacement of the inner canthi of eyes (dystopia canthorum). Based on genetic and clinical criteria, four types of WS have been recognised (www.omim.org, accessed on 11 April 2024, OMIM: PS193500; [10]).

Additionally, other variants in *PAX3*, *SOX10*, *MITF*, *EDNRB,* and *KIT* have been associated with white spotting or a white coat in farm and companion animal species including cattle, buffaloes, pigs, sheep, horses, donkeys, goats, dogs, and cats (omia.org, accessed on 11 April 2024). In addition to white spotting or a white coat, some of these variants are associated with heterochromia or blue eyes, deafness, or symptoms resembling WS in humans. In cats, two *KIT* retroviral insertions underlie the *w^S^* and *W* alleles described at the white locus. No deleterious phenotype has been described to occur in animals with the *w^S^* allele, whereas deafness is associated with white fur governed by the *W* allele. Additionally, both alleles have been shown to produce heterochromia or blue eyes in some individuals [3]. As mentioned before, blue eyes are largely appreciated by feline breeders and owners. Thus, when a dominant phenotype characterised by heterochromia or two blue eyes and minimal white spotting was found in New Mexico (USA) in the 1980s, a feline breed named Ojos Azules was developed using this new trait. The breed was accepted for registration by TICA in 1991. Controlled breeding studies conducted by Solveig Pfleuger, MD, and others identified white kittens with cranial deformities born to two blue-eyed parents and reduced the enthusiasm for the breed. WS genes including *PAX3* were considered viable candidate genes for the condition. Abnormal kittens were supposed to be homozygous for the mutant allele. Finally, the incomplete penetrance of the blue eye phenotype confirmed by the birth of blue-eyed kittens born to two non-blue-eyed parents led to the decline of the breed. Today, the Ojos Azules is considered extinct (http://messybeast.com/DBE-ojos-azules.htm, accessed on 11 April 2024). In the mid-1990s, in Kazakhstan, cats resembling the American Ojos Azules were found in the streets. A new feline breed named Altai was created using one of these blue-eyed outbred Kazakh cats named Fyodor. The blue eye and minimal white spotting phenotype is dominant and was named DBE for dominant blue eyes. In contrary to the American Ojos Azules, breeders discovered that homozygous mutant kittens were viable and showed no morphological defects. These kittens had large white spots or white fur, and some of them appeared to be deaf. Altai breeders also found that certain cats carry the DBE variant but do not express the blue eye phenotype. These cats have been named latent cats. Altai cats still exist, and the breed is developed under the framework of the World Cat Federation (WCF: http://wcf.info, accessed on 11 April 2024) registering body (http://messybeast.com/DBE-altai.htm, accessed on 11 April 2024). In the following years, other outbred cats with minimal white spotting and blue eyes were found in Kazakhstan and Russia and were used by breeders. Especially, the Topaz breed was developed using two DBE founding cats (named Roxi and Seymour) of different origins and believed to carry two distinct DBE variants, one of which is the original Altai DBE variant. The DBE trait has spread into feline breeds on every continent, and today, several DBE variants are thought to segregate into DBE lines, some of which have identified origins, while others remain elusive (http://messybeast.com/blue-eye-breeds.htm, accessed on 11 April 2024). Previously, we identified two *PAX3* variants associated with two DBE traits in a Dutch Maine Coon lineage [11], in the Celestial breed, and in certain Maine Coon and Siberian lineages [12], respectively. Here, we report the identification of a third *PAX3* variant associated with DBE, and we review the presence of the three *PAX3* variants in 14 DBE feline breeding lines, including the Altai line.

## 2. Materials and Methods

### 2.1. Animals

A total of 177 cats was included in the genetic study, including 117 DBE cats from various lineages. They were sampled in Europe, Ukraine, Russia, the UK, and the USA from September 2016 to April 2024 and included individuals from the following breeds: British shorthair and longhair (*n* = 53, including 31 DBE cats,), Altai (*n* = 17 DBE cats), Celestial (*n* = 24, including 22 DBE cats), mixed-breed cats (*n* = 14, including 5 DBE cats), Siberian (*n* = 6, including 4 DBE cats), Maine Coon (*n* = 28, including 25 DBE cats), Sphynx (*n* = 12, including 3 DBE cats), Persian and Exotic shorthair (*n* = 7, including 5 DBE cats), Ragdoll (*n* = 4 DBE cats), Chinese Tank (*n* = 1 DBE cat), domestic shorthair (*n* = 2), Chartreux (*n* = 2), Siamese (*n* = 2), Devon Rex (*n* = 1), Donskoy (*n* = 1), Birman (*n* = 1), Turkish Angora (*n* = 1), and Bengal (*n* = 1). All cats were included following their owners’ consent. Non-invasive buccal swabs were sent back directly by the owners or collected by a veterinarian. One cat was spontaneously presented to his regular veterinarian. He was clinically evaluated prior to the blood collection. Pedigrees and clinical data (brainstem auditory evoked response, BAER, and test results) were collected from the owners.

### 2.2. Ethics Statement

All animals were client-owned cats on which no harmful invasive procedures were performed, so there was no animal experimentation according to the legal definition in Europe (Subject 5f of Article1, Chapter I of the Directive 2010/63/UE of the European Parliament and of the Council).

In one cat, blood was obtained as part of a routine clinical procedure for diagnostic purposes, at the request and with the consent of the owner.

The DNA from other cats was obtained non-invasively (cheek swabs) specifically for this study or for other studies, at the request of their owners who consented that their cats’ DNA could be used in research projects aimed at improving feline knowledge, health, and welfare. The project was evaluated by the Ethics Committee of VetAgro Sup and received the agreement number 2042.

### 2.3. SNP Genotyping, Genome-Wide Association Study, and Genome Sequencing

DNA was extracted from whole blood and buccal swabs according to the manufacturers’ protocols, using either a Maxwell^®^ 16 Instrument (Promega Corporation, Madison, WI, USA) or the NucleoSpin 96 Tissue DNA Kit (Macherey-Nagel EURL, Hoerdt, France).

Sixty-six cats were genotyped using the Illumina Infinium iSelect 63k Cat DNA SNP genotyping array (Illumina, Inc., San Diego, CA, USA). For each cat, 700 ng of genomic DNA was sent to the Neogen laboratory (www.neogen.com, accessed on 21 April 2024). The arrays were processed according to the manufacturer’s protocol. The SNP genomic positions were inferred according to the updated SNP manifest for the Illumina Feline 63k SNP array [13]. The SNP genotyping rate and minor allele frequency were assessed using PLINK v1.90 software ([14]; https://zzz.bwh.harvard.edu/plink/, accessed on 10 June 2023). SNPs with an MAF < 5%, genotyping rate < 95%, and individuals genotyped for <95% of SNPs were excluded from downstream analyses. Case-control association analyses were performed using PLINK. The *p* values were corrected according to the Bonferroni procedure. Manhattan plots of the results were generated using the ggman R package for Manhattan plots and R 3.4.3 (www.r-project.org/, accessed on 10 June 2023).

For whole-genome sequencing, a PCR-free DNA library with a 400 bp insert size of a DBE cat was prepared by IntegraGen (IntegraGen, Evry, France) using a NEBNext Ultra II DNA Library Prep Kit for Illumina (www.neb.com, accessed on 21 April 2024). IntegraGen generated 150 bp paired-end reads on an Illumina NovaSeq 6000 instrument (30X coverage). Mapping and alignment were performed using the Burrows–Wheeler Aligner (BWA) tool and the Felis_catus 9.0 genome reference excerpted from Ensembl (www.ensembl.org, accessed on 10 June 2023) by IntegraGen. Variant calling and filtering were performed by IntegraGen using GATK 3.8 [15] and MANTA v1.6.0 [16].

### 2.4. PAX3 Sequencing and Variants Genotyping

*PAX3* reference sequence was collected from Ensembl (www.ensembl.org, accessed on 5 March 2024; feline *PAX3* gene (ENSFCAT00000018878.5, annotation release 104 for Felis_catus 9.0 genome assembly)). PCR and sequencing primers were designed using Primer3 [17]. Exons and intron–exon boundaries were amplified for each *PAX3* exon using primers from Table S2 (Supplementary Table S2). The sequences were amplified individually for each cat from 100 ng of their genomic DNA according to the manufacturers’ protocol, with GoTaq G2 Hot Start Polymerase, 2 mM MgCl_2_, 0.5µM of each primer, and 35 cycles (Promega Corporation, Madison, WI, USA). Four hundred ng of each PCR amplicon was sent to Eurofins (Eurofins Genomics, Koln, Germany), purified, and Sanger sequenced in both the forward and reverse directions. Electropherograms were manually inspected with Chromas Lite (Technelysium Pty Ltd., South Brisbane, Australia).

For NC_018730.3:g.206975776_206975777insN[433] and NC_018730.3:g.206974029_206974030insN[395] genotyping, partial sequences of *PAX3* intron 4 were amplified using PCR using 20 to 100 ng of genomic DNA, an annealing temperature of 60 °C, the GoTaq G2 Hot Start Polymerase, 2 mM MgCl_2_, 0.5 µM of each primer, and 35 cycles (Promega Corporation, Madison, WI, USA). The primers and product sizes are shown in Table S2 (Supplementary Table S2). PCR products were resolved using 3% agarose-gel electrophoresis. For verification, gel purification (Macherey-Nagel™ NucleoSpin™ Gel and PCR Clean-up Kit) and Sanger sequencing, or direct sequencing, was performed on a batch of samples using PCR primers as the sequencing primers. Gel-purified PCR amplicons or whole PCR products were sent to Eurofins (Eurofins Genomics, Koln, Germany) and Sanger sequenced in both the forward and reverse directions. Electropherograms were manually inspected with Chromas Lite (Technelysium Pty Ltd., South Brisbane, Australia). Multiple alignments were performed using Multalin ([18]; http://multalin.toulouse.inra.fr/, accessed on 5 March 2024; BLOSUM-62 and identity matrix).

To characterise the insertion in *PAX3* intron 4, we used BLAST (https://blast.ncbi.nlm.nih.gov/Blast.cgi, accessed on 5 March 2024). The alignment of the RD-114 virus sequence (GenBank: AB559882.1) with the NC_018730.3:g.206975776_206975777insN[433] sequence was performed using Multalin ([18]; http://multalin.toulouse.inra.fr/multalin/, accessed on 5 March 2024).

### 2.5. Accession Numbers

SNP genotyping data were deposited at OSF (https://osf.io/k798c/, accessed on 28 March 2024). A partial genomic sequence of *PAX3* intron 4 from a DBE cat (*Felis catus*) with the insertion was submitted to GenBank; the accession number is GenBank ID: PP332291. A whole-genome sequence of the DBE cat was submitted to SRA. The accession numbers are BioProject ID: PRJNA1073398 and BioSample ID: SAMN39921596.

## 3. Results

### 3.1. Several Breeding Lines Segregate DBE

We collected phenotypical and genealogical data along with cheek-cell samples for 117 DBE cats from 14 different lineages (Table 1) and mixed lines.

**Figure 1:**
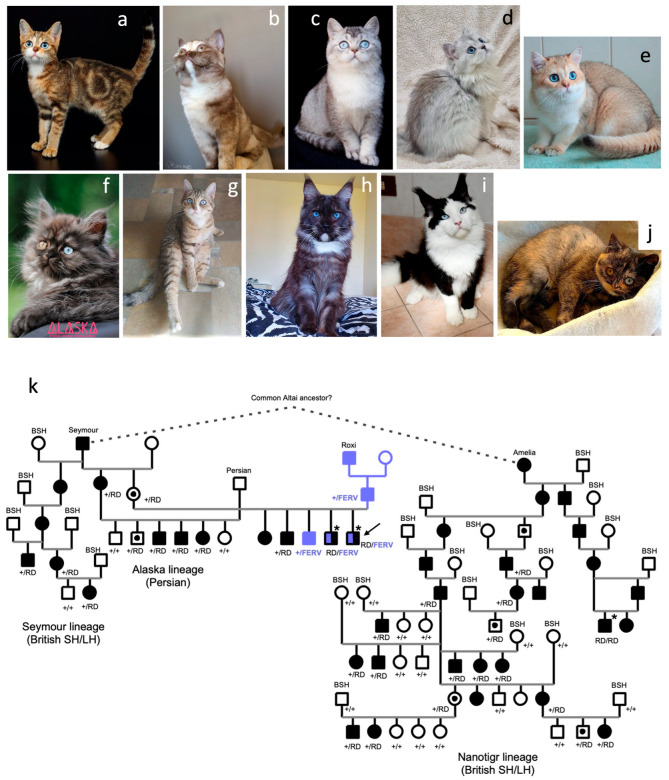
DBE is present in various lineages of cats. (**a**–**j**) DBE phenotype in some feline breeding lines. (**a**) Altai cat. (**b**) Mixed-breed cat, daughter of the founding cat named Seymour. Note the sectorial heterochromia of both eyes. (**c**) British cat from the Nanotigr lineage. (**d**) British cat from the Igor lineage (**e**) British cat from the Nadeya lineage. (**f**) Persian–mixed cat from the Alaska lineage. (**g**) Founding female (named Marusya) from the Cyrridwen lineage. (**h**) Maine Coon cat from the Topaz lineage. All these cats were born to a DBE parent with minimal white spotting and to a non-DBE, non-white-spotted parent. They were therefore assumed not to be carrier of the *w^S^* allele. (**i**) Maine Coon cat from the Dutch lineage. Note the large amount of white: this cat was tested heterozygous *w^S^/w^+^* for the white spotting locus. (**j**) Celestial kitten. Note the small white spot on the chin. (**k**) Partial pedigree tree of DBE lineages. Circles represent females; squares represent males. DBE cats with heterochromia or two blue eyes are depicted with fully filled symbols. The two Topaz founding cats are shown with their names (Roxi and Seymour) according to http://messybeast.com/blue-eye-breeds.htm, accessed on 11 April 2024. Roxi, the Celestial founding cat, and one of its litters are shown in blue and black. The cat that was whole-genome sequenced is shown with an arrow. Stars point out white-coated and deaf cats born to two DBE parents. Genotypes for the RD-114 LTR insertion are shown (+/+: wild-type; +/RD: heterozygous for the RD-114 LTR insertion; RD/RD: homozygous for the RD-114 LTR insertion). Genotypes for the FERV1 LTR insertion in the Roxi family are shown (+/+: wild type; +/FERV: heterozygous for the FERV1 LTR insertion; RD/FERV: compound heterozygous for both insertions). Latent cats are shown with a dot in their symbol. This pedigree tree was concordant with an autosomal dominant inheritance for DBE. A shared RD-114 LTR insertion among the Altai and the Seymour and Nanotigr British lineages suggests a common ancestor for these three breeding lines. Amelia, the DBE daughter of the founding male of the Nanotigr lineage (named Oliver) is shown. SH: shorthair, LH: longhair, BSH: British cat (shorthair or longhair).

All the DBE cats born to a DBE parent and a non-DBE parent without white spotting shared common phenotypic features: one or two blue eyes or sectorial heterochromia, minimal white spotting, or an undetectable white spot (Figure 1a–j).

One of these 14 lineages was created from the original blue-eyed Altai breed (Figure 1a). Among the seventeen cats from this lineage, two cats were born to two DBE parents and showed a white fur with a coloured tail for one cat and a white fur with a coloured spot on the back for the other cat (Supplementary Figure S1). The remaining 15 cats were born to a DBE parent and a non-DBE parent and showed minimal white spotting. The two almost-white cats had a normal hearing status according to the breeder but as no BAER test (brainstem auditory evoked response) was possible, we were unable to exclude unilateral deafness.

Four breeding lines were developed with the British shorthair and longhair genetic background (Figure 1b–e). Three used an outbred domestic shorthair cat to introduce the DBE trait. For one line, the founder cat was Seymour (Figure 1b), one of the two Topaz-breed founding studs. Two other lines have used DBE founding cats with unrecorded origins: a cat named Igor for the “Igor” line (Figure 1c) and another stud named Oliver for the “Nanotigr” line (Figure 1d). From the Nanotigr line, we obtained data and cheek swabs for a kitten born to two DBE parents. This cat had white fur with a coloured tail. According to the owner, this cat was deaf. From the Igor lineage, breeders reported three deaf cats among more than 50 DBE kittens that were produced. All three cats were born to a DBE parent and a non-DBE parent and had white spotting on their head. The last lineage experienced a spontaneous DBE variant, according to the breeder (“Nadeya” line, Figure 1e). In this Nadeya line, several DBE-to-DBE matings were conducted, none resulting in white kittens or bicolour kittens with a large visible amount of white in the fur. Three deaf cats were recorded among tens of DBE kittens that were produced. Two of them had minimal white spotting and were born to two DBE parents. The last one had a white spot on its head and was born to a DBE parent and to a non-DBE parent (a purebred British shorthair cat with full coloured fur and copper eyes).

Two breeding lines were created using Persian and Exotic shorthair crosses (Figure 1f,g). The first one, developed in France, used Seymour as the founding cat (“Alaska” lineage, Figure 1f), and the second one, developed in Russia, used an outbred female named Marusya, with unknown origins (“Cyrridwen” lineage, Figure 1g).

Four Ragdoll cats with a DBE phenotype were included in the study. They belonged to an American lineage founded by a male born to Seymour (Table 1).

The pedigree data analysis revealed no DBE founding cat in the genealogy of the three DBE Sphynx cats included in the study. Similarly, four Siberian cats with an unknown origin for DBE were recruited (Table 1).

We collected data from three distinct Maine Coon lines with DBE (Figure 1h,i). The first one was founded using a Topaz stud (Figure 1h), whereas the second one segregated a *PAX3* nonsense variant that arose in the Maine Coon genetic background (Dutch lineage, Figure 1i, [11]). The third lineage (Pillowtalk line) was of unknown origin (Table 1). In the Maine Coon breed, a litter born to two DBE parents was reported. The mother was from the Topaz line and the father was from the Dutch line. This mating produced a white kitten that died just after birth and showed an enlarged head and contracted and abnormal limbs. A fourth Maine Coon line with DBE has been described (Nahal line, Table 1, http://messybeast.com/blue-eye-breeds.htm, accessed on 25 April 2024), but no data were available for it.

Finally, we included Celestial cats in the study (Figure 1j). In this breed, founded with the outbred DBE stud named Roxi, we recently identified a FERV1 LTR (long terminal repeat) insertion in *PAX3* intron 4 [12]. In this breed, a single DBE-to-DBE mating resulted in a litter including a white kitten that died just after birth from a cleft palate [12]. According to the breeder, this kitten also showed limb abnormalities.

The collection of genealogical data allowed for the pedigree tree drawing for three lineages: British and Persian lineages based on the Seymour founder and the British Nanotigr lineage (Table 1, Figure 1k). We also identified a mixed litter born to two DBE parents that belonged to the Seymour lineage for the mother and the Roxi lineage for the father. In this five-kitten litter, two males were born, with a white coat for the first one and an almost-white coat for the second one, and revealed to be deaf (Figure 1k). All genealogical data were concordant with an autosomal dominant inheritance pattern for DBE, in accordance with the breeder’s reports.

### 3.2. A Second PAX3 Insertion Is Associated with DBE and Spread in Feline Lines

Two DBE cats of each lineage were genotyped for the previously reported variants (PAX3:c.937C>T nonsense variant and *PAX3* NC_018730.3:g.206974029_206974030insN[395] LTR insertion [11,12]) except for the Cyrridwen lineage, for which only one cat was available and genotyped. The nonsense variant was present in the two Maine Coon cats from the Dutch lineage but absent in the cats from the 13 other lineages. The LTR insertion was identified in the two cats from the Celestial breed, in the two Siberian cats, and in the two Maine Coon cats from the Topaz lineage. The other DBE cats from the remaining 11 lineages were wild type for the insertion.

**Figure 2:**
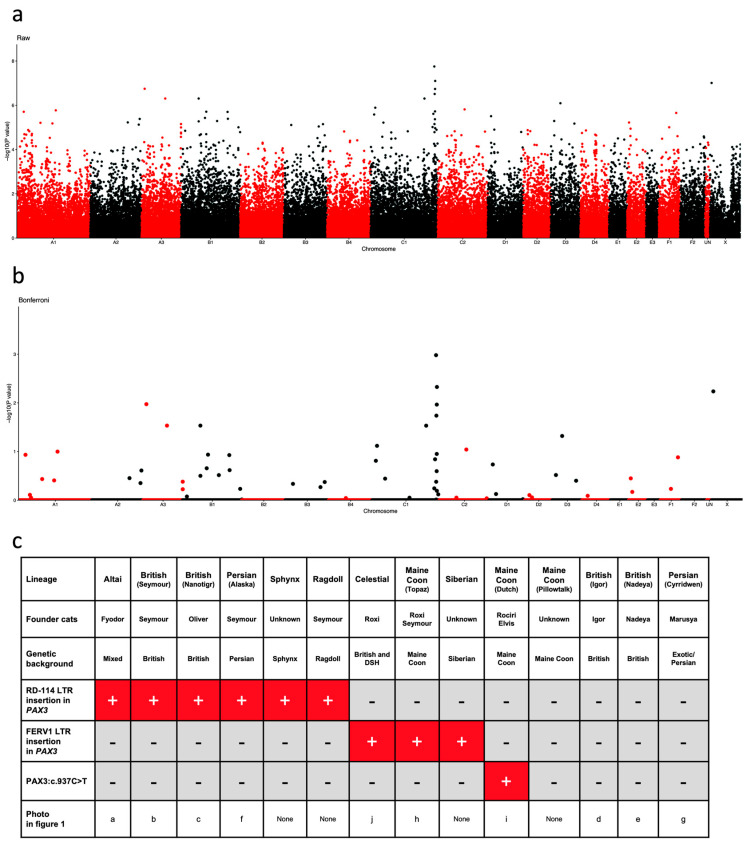
At least three *PAX3* variants are associated with DBE. (**a**,**b**) Manhattan plots of the GWAS. The plots represent the −log10 *P_raw_* and −log10 *P_Bonferroni_* values of each SNP included in the case–control association study. The association study compared 29 DBE cats with 37 control cats. A suggestive association with chromosome C1 was detected. The SNP with the highest association was chrC1.233232798!208720404 at position 208,720,404, with a *P_raw_* value of 1.76 × 10^−8^ and *P_Bonferroni_* value of 1.05 × 10^−3^ (Table S1). UN: unknown. (**c**) Presence (+) and absence (-) of the two FERV1 LTR and RD-114 LTR insertions in *PAX3* intron 4 and the PAX3:c.937C>T exon 6 variant in 14 DBE breeding lines. As shown in Figure 1, the shared RD-114 LTR insertion among the Seymour and Nanotigr British lineages and the Altai breed suggests a common ancestor for these three breeding lines. Four breeding lines do not carry any of the three *PAX3* variants, confirming genetic heterogeneity of the DBE trait in cats. DSH: domestic shorthair.

To search for other variants in British shorthair and longhair cats with DBE, 29 DBE cats and 37 control cats were genotyped using the Illumina Feline 63k SNP array. These cats were mainly from the Nanotigr and Seymour lineages. None of the control cats exhibited white spotting. A total of 60,611 SNPs yielded usable results (minor allele frequency MAF > 5%, genotyping rate > 95%). All 66 cats had genotyping rates > 95%, and all were conserved for the analysis. Following basic case–control analysis, the genomic inflation factor was 2.1, and the 20 highest significant associations were identified for 20 SNPs, among which 9 markers were located on chromosome C1 (Figure 2a, Supplementary Table S1).

After Bonferroni correction of the *P_raw_* values for multiple tests, a single SNP located at position 208,720,404 on chromosome C1 had a significant *P_Bonferroni_* value (Figure 2b, Supplementary Table S1). Six SNPs from chromosome C1 were located between position 205,596,490 bp and position 211,885,885 bp (Supplementary Table S1) according to Felis_catus 9.0 reference genome. This region contains the strong candidate gene *PAX3* (chromosome C1: 206,906,912 bp–207,004,190 bp, Felis_catus 9.0). Exons and exon–intron boundaries from *PAX3* were sequenced in two British DBE cats from the Seymour lineage, in one of the two white and deaf cats from the mixed litter born to two DBE parents belonging to the Roxi and Seymour lineages (Figure 1k), a DBE Celestial cat from the Roxi lineage, and a DBE British cat from the Nadeya lineage and compared to the reference feline sequence (transcript ENSFCAT00000018878.5, Supplementary Table S2). Nine synonymous variants were identified (Supplementary Table S3).

The whole-genome sequence of one of the two white and deaf cats from the mixed litter born to two DBE parents was analysed (Roxi and Seymour founding cats, Figure 1k) to screen for non-coding variants in *PAX3* regulatory sequences and across the candidate region (Supplementary Table S4). Paired-end reads (2 × 150 bp) were collected from a shotgun DNA fragment library achieving genome-wide coverage of 30X. The variants were called against the Felis_catus 9.0 reference genome. Two candidate variants were identified. The first variant was NC_018730.3:g.206975776_206975777insN[433] located in the intronic region of *PAX3* (Supplementary Table S4). This variant consisted in a 433 bp insertion at position 206,975,776 in *PAX3* intron 4. The second variant, namely NC_018730.3:g.206974029_206974030insN[395] consisted in the 395 bp insertion at position 206,974,029 in *PAX3* intron 4 that was previously associated with DBE in the Celestial breed (Supplementary Table S4, [12]).

As control cats from various breeds have previously been genotyped as wild type for the NC_018730.3:g.206974029_206974030insN[395] variant [12], 60 non-DBE control cats were genotyped from various breeds (British, Bengal, Birman, Celestial, Chartreux, Devon Rex, Donskoy, Maine Coon, Persian, Siamese, Siberian, Sphynx, Turkish Angora, domestic shorthair, mixed breed) for the NC_018730.3:g.206975776_206975777insN[433] variant. None carried the insertion (Table 2).

Overall, 117 DBE cats were genotyped for this variant, representing 14 lineages or being from mixed lines founded by the Seymour and Roxi sires (Table 2). The variant was heterozygous in all DBE cats from the Altai breed (*n* = 15); the Seymour British, Persian, and Ragdoll lineages (*n* = 13); the Nanotigr British lineage (*n* = 13); the three Sphynx with DBE of unknown origin; the Chinese Tank (Munchkin–British mixed cat) from a mixed Seymour and Roxi breeding line; and in the two white and deaf cats and one of their littermates from the mixed litter born to two DBE parents (Roxi and Seymour lineages, Figure 1k). This variant was heterozygous in five latent cats (cats that carry the DBE variant but do not express the blue eye phenotype) from Seymour and Nanotigr lines (Table 2, Figure 1k). Additionally, three homozygous cats in the Atlai (*n* = 2) and Nanotigr (*n* = 1) lines were identified, and all three were white or almost-white cats and born to two DBE parents (Table 2). Eleven DBE cats from a mixed Seymour and Roxi genetic background were genotyped as wild type for the variant (Topaz Maine Coon cats: *n* = 10; mixed-breed cat: *n* = 1; Table 2). Finally, DBE, white, and latent Celestial cats (*n* = 22), along with all DBE Siberian cats (*n* = 4), the Persian cat from the Cyrridwen line, the British cats from the Nadeya (*n* = 2) and Igor (*n* = 6) lines, and the Dutch (*n* = 13) and Pillowtalk (*n* = 2) lines of Maine Coon cats were genotyped as wild type for the NC_018730.3:g.206975776_206975777insN[433] variant (Table 2).

The segregation of the NC_018730.3:g.206975776_206975777insN[433] variant in the lineages for which pedigree data were available was consistent with an autosomal dominant inheritance, and we observed a perfect genotype–phenotype correlation in these lineages (Figure 1k).

We completed the genotyping assay by testing DBE cats for the two *PAX3*:c.937C>T and NC_018730.3:g.206974029_206974030insN[395] variants previously identified (Supplementary Table S5). In conclusion, *PAX3*:c.937C> was restricted to the Dutch Maine Coon cats but the NC_018730.3:g.206974029_206974030insN[395] and NC_018730.3:g.206975776_206975777insN[433] variants were present in various DBE lineages. Finally, 4 of the 14 lineages we studied lacked a known DBE variant (Figure 2c, Supplementary Figure S4).

The NC_018730.3:g.206975776_206975777insN[433]-inserted sequence consisted of an element that demonstrated the highest level of identity to an LTR (long terminal repeat) from an RD-114 feline endogenous retrovirus (Supplementary Figures S2 and S3) and is located in the fourth intron of *PAX3*, which is known to contain five regulatory elements named CNE1 to CNE5 (conserved non-coding element, [12,19]). The RD-114 LTR insertion at position 206,975,776 on chromosome C1 lied in the vicinity of these CNEs, especially CNE3 and CNE4.

## 4. Discussion

### 4.1. Genetic Heterogeneity of Feline DBE

Since the discovery of Fyodor, the DBE founding cat of the Altai breed in Kazakhstan in the 1990s, several other DBE cats have been recorded by the *Cat Fancy*. Some individuals were rescued streets cats, some have appeared in established breeds with pedigree data, and some were born from crossbreeding through unregistered matings. Data and samples from 14 of around 20 recorded DBE breeding lines to date (http://messybeast.com/blue-eye-breeds.htm, accessed on 11 April 2024) were collected. Two *PAX3* variants associated with DBE in some feline lineages, the *PAX3*:c.937C>T nonsense variant [11] and the *PAX3* NC_018730.3:g.206974029_206974030insN[395] LTR insertion [12], were previously identified. The NC_018730.3:g.206975776_206975777insN[433] LTR insertion constitutes a third variant associated with DBE. All three variants are located in the *PAX3* gene and arose independently.

The *PAX3*:c.937C>T is restricted to a Dutch Maine Coon lineage (Figure 2c) known by breeders as the “Rociri Elvis” lineage (http://messybeast.com/DBE-maine-coon.htm, accessed on 11 April 2024). This allele, named *DBE^RE^* (*Rociri Elvis Dominant Blue Eye*), was shown to be associated with deafness in heterozygous cats. For this variant, no homozygous mutant animals were reported, indicating that the homozygous state of this allele is likely lethal [11].

The NC_018730.3:g.206974029_206974030insN[395] variant (*DBE^CEL^*) was found in the Celestial breed, and in this breed, DBE cats were BAER tested prior to registration, and the variant has not been associated with deafness [12]. The Celestial breed was created in France using an outbred male born to the Roxi sire. The *DBE^CEL^* allele was also present in the Maine Coon lineage founded by a Topaz cat. The Topaz breed was created using Roxi and Seymour, thus mixing two DBE lineages. In the Topaz-based Maine Coon line, only the *DBE^CEL^* variant from the Roxi lineage was identified. To date, we identified a single Maine Coon line founded by a unique Topaz cat. We assume that this Topaz cat transmitted the *DBE^CEL^* allele to its descendants. However, the NC_018730.3:g.206975776_206975777insN[433] variant of the Seymour lineage could be present in other DBE Maine Coon cats coming from a Topaz founder. The *DBE^CEL^* variant was also present in the four Siberian cats included in the study. These cats were all related to a single DBE female declared born to two purebred Siberian cats: a brown tabby and white male with copper eyes and a brown tabby and white female with green eyes. Therefore, the origin of the DBE trait in this lineage was unknown, but it is very likely a crossbreeding introduced the DBE trait. Haplotype analysis could help reveal the origin of the trait in this line.

The new RD-114 LTR NC_018730.3:g.206975776_206975777insN[433] variant was identified in the Altai breed, in the Alaska Persian line, in a Ragdoll line derived from Seymour, and in two British shorthair and longhair lines founded using Seymour (Seymour lineage) and a stud named Oliver with an unknown origin (Nanotigr lineage), respectively. The Altai breed resulted from a single founder named Fyodor, who lived in Ust-Kamenogorsk in Kazakhstan (http://messybeast.com/DBE-altai.htm, accessed on 11 April 2024). Seymour was described as a domestic shorthair cat found in Russia (http://messybeast.com/blue-eye-breeds.htm, accessed on 11 April 2024). Oliver originated from a Russian cattery and was registered as a British shorthair. He sired a black and silver shaded female with odd eyes named Amelia Nanotigr, the ancestor of all the British shorthair and longhair cats from the Nanotigr lineage we studied. As the origins of Seymour and Oliver are unknown, it is likely that both lines descend from the first Altai cats and are related to Fyodor (Figure 1k). Additionally, the NC_018730.3:g.206975776_206975777insN[433] variant appears to be associated with the original Altai DBE trait. Thus, we propose that this variant represents the *DBE^ALT^* (*Altai dominant blue eye*) allele. This allele also represents the *Seymour DBE* allele, contrary to what had been hypothesised. Indeed, Roxi was supposed to be an Altai cat, and Seymour, a distinct DBE founder (http://messybeast.com/blue-eye-breeds.htm, accessed on 11 April 2024). Our results contradict this hypothesis. Roxi transmitted a peculiar DBE variant: the *DBE^CEL^* allele. He was probably not an Altai cat. Seymour transmitted the Altai *DBE^ALT^* allele; he was probably an Altai cat.

In the five-kitten mixed litter born to two DBE parents (Figure 1k), we had DNA for both parents and four kittens. The latent dam from the Seymour line was heterozygous for the *DBE^ALT^* allele. The DBE sire was heterozygous for the *DBE^CEL^* allele. One DBE kitten was heterozygous for the *DBE^ALT^* allele, while the other DBE kitten was heterozygous for the *DBE^CEL^* allele. The two white and deaf kittens were compound heterozygous for both *DBE^CEL^* and *DBE^ALT^* alleles (Figure 1k). This litter demonstrated that cats with these two DBE alleles are viable but are at high risk of deafness. Breeders are warned that mating two DBE cats from the Roxi and Seymour origins may be deleterious. The only kitten born to two Celestial DBE parents and homozygous for the *DBE^CEL^* allele was white and died at birth [12]. This type of mating should also be avoided.

Finally, the four lineages we studied still lack a DBE variant. The origin of DBE in the Pillowtalk lineage of Maine Coon cats is unknown. The Cyrridwen Persian/Exotic shorthair lineage has been founded using a Russian outbred female named Marusya, whose origin is unknown. The British shorthair and longhair lineage coming from Igor, a DBE male found in Kazakhstan, does not share any variant with the Altai or Roxi lineages, whereas it had been assumed that Igor was an Altai cat (http://messybeast.com/DBE-azure-dream.htm, accessed on 11 April 2024). Also, in the Nadeya British lineage that is hypothesised to have a *de novo* DBE variant, none of the three *PAX3* variants were identified.

### 4.2. Three Spontaneous Variants in the PAX3 Gene

In the domestic cat, dominant white (*W*) and white spotting (*w^S^*) alleles were both identified in the non-coding region of *KIT* [3]. A full-length feline endogenous retrovirus (FERV1) insertion in the *KIT* first intron underlies *w^S^*, whereas a FERV1 LTR insertion underlies *W*. The LTR element exhibited complete sequence identity between the *W* and *w^S^* alleles. Thus, the most plausible hypothesis was that the integration of the full-length FERV1 had appeared first, and that the *W* allele has been produced by recombination between the two LTRs of the integrated FERV1, generating a single LTR [3].

LTR insertions are found for many classes of endogenous retroviruses, and they outnumber their full-length ancestral retrovirus progenitors [20]. As no coding variant was identified in the strongest candidate gene, namely *PAX3*, insertions and deletions (structural variants) were considered, because an upstream enhancer that mediates the hypaxial somite expression of *PAX3* has been identified more than 6 kb upstream of the transcription start site, and because the 1.6 kb upstream region of *PAX3* was shown to contain two evolutionarily conserved elements that are critical for its expression. Additionally, conserved non-coding elements were identified within the fourth intron of *PAX3* [19,21]. Three variants are now associated with DBE: a *PAX3*:c.937C>T nonsense variant [11] and two distinct LTR insertions in *PAX3* intron 4. These two LTRs are derived, respectively, from FERV1 for the *DBE^CEL^* allele [12] and from an RD-144 virus for the *DBE^ALT^* variant (Supplementary Figures S2 and S3). Interestingly, in the domestic cat, three retroviral elements cause a white coat, white spotting phenotypes, and eye hypopigmentation by inserting in the vicinity of regulatory sequences from two genes involved in melanoblast biology [3,4,12]. Retroviral elements have been shown to represent approximately 4% of the assembled feline genome [22]. As observed for the FERV1 *KIT* insertion, the two FERV1 and RD-114 LTR insertion sites observed in *PAX3* are unusual compared to the pattern of ERV insertions reported in the human genome. Indeed, ERVs have been frequently found in intergenic regions and are rarely found within intronic regions or in the vicinity of genes [3,23].

### 4.3. PAX3-Related DBE in Cats Share Some Common Features but Not All with PAX3-Related Waardenburg Syndrome

The two LTR inserted into *PAX3* intron 4 are located near the CNE2, CNE3, and CNE4 conserved sequences that were shown to be regulators of *PAX3* expression [12,19]. *PAX3* encodes a transcription factor shown to be a key regulator of *MITF,* which is involved in melanocyte development and responsible for pigment–cell-specific transcription of the melanogenesis enzyme genes [24]. Variants in *PAX3* and *MITF* have been associated with auditory–pigmentary phenotypes in animal species (www.informatics.jax.org/allele/MGI:1856173, accessed on 11 April 2024; OMIA:001688-9796; [8,9,25,26,27]) and in auditory–pigmentary Waardenburg syndromes (WS) in humans (OMIM: PS193500; [10]). Two types of *PAX3*-related WS have been identified: WS type 1 and type 3. WS type 1 is characterised by pigmentation defects of the hair, skin, and eyes; congenital deafness; and the lateral displacement of the inner canthi of the eyes (dystopia canthorum). *PAX3*-related WS type 3 is also characterised by the presence of this dystopia canthorum and upper limb abnormalities, whereas *PAX3*-unrelated WS types 2 and 4 lack dystopia canthorum. Besides depigmentation and deafness, WS type 4 also includes Hirschsprung disease (aganglionic megacolon, OMIM: 193500). WS type 2 has been associated with variants in *MITF, SOX10*, and *KITLG* (OMIM: 193510), and WS type 4 with variants in *EDNRB, EDN3*, and *SOX10* (OMIM: 277580). Feline DBE share some features with WS: hypopigmentation of the hair, skin, and eyes. Among DBE cats carrying a non-coding *PAX3* variant, bilateral deafness was observed in homozygous *DBE^ALT^*/*DBE^ALT^* cats or in compound heterozygous *DBE^CEL^*/*DBE^ALT^* cats. Deafness was also reported in three cats out of tens of kittens produced in the Nadeya line and in three cats among more than fifty kittens that were produced in the Igor line (unknown DBE underlying variants, Table 1). Among Maine Coon cats carrying the *PAX3*:c.937C>T nonsense variant (*DBE^RE^* allele), unilateral and bilateral deafness has been confirmed using the BAER test in heterozygous cats [11]. Thus, in feline *PAX3*-related DBE, deafness appears to depend on the molecular defect. While non-coding variants do not lead to deafness in heterozygous individuals, the cumulative impact of two mutant alleles in homozygous animals may compromise PAX3 function. Indeed, bilateral deafness has been observed in cats that are homozygous for the DBE^ALT^/DBE^ALT^ alleles, as well as in compound heterozygous DBE^CEL^/DBE^ALT^ cats. However, additional research is needed to evaluate the prevalence of deafness among the various DBE lineages, especially of unilateral deafness, which cannot be reliably assessed without BAER testing. In the Maine Coon background, some DBE cats have been reported with dystopia canthorum, but to date, we cannot link this defect to DBE alone or to the interaction between DBE and a specific face morphology that has been accentuated by selection in certain Maine Coon lines and has resulted in a large muzzle, big ears, and small sunken eyes. Dystopia canthorum was not observed in adult DBE cats from the Altai, Celestial, British (Figure 1), Sphynx, and Siberian genetic backgrounds.

Only a limited number of kittens born to two DBE parents have been reported. Homozygous *DBE^ALT^*/*DBE^ALT^* are viable and show no abnormalities except deafness for some of them. A single homozygous *DBE^CEL^*/*DBE^CEL^* kitten and a single putative compound heterozygous *DBE^CEL^*/*DBE^RE^* kitten have been reported. They both showed limb abnormalities and contractures, an abnormal head morphology, and they died at birth. In humans, limb abnormalities and contractures have been reported in WS type 3 due to dominant variants but also in homozygous or compound heterozygous children from families with *PAX3* missense variants usually associated with WS type 1 [28,29,30]. Thus, the feline *PAX3*-related DBE studied here cannot be reduced to a feline WS [10]. It can be defined as an auditory–pigmentary syndrome that shares some common features with *MITF*-related WS type 2 (absence of dystopia canthorum), *PAX3*-related WS type 1 (low prevalence of deafness), and *PAX3*-related WS type 3 (limb abnormalities and contractures in certain cases of homozygous or compound heterozygous kittens). This auditory–pigmentary syndrome (OMIA:001688-9685) is phenotypically and genetically heterogenous, and certain DBE-to-DBE matings may produce abnormal kittens. In this context, breeders are encouraged to avoid DBE-to-DBE matings and the mix of DBE lines and to test their breeding stocks for the known *PAX3* variants to avoid at-risk matings.

## 5. Conclusions

In conclusion, a third *PAX3* variant associated with the DBE phenotype in the Altai breed and other feline lineages was identified. The NC_018730.3:g.206975776_206975777insN[433] variant, named *DBE^ALT^* allele, is located within the fourth intron of *PAX3* in the vicinity of non-coding conserved elements involved in *PAX3* expression. It expands the known spectrum of *PAX3* variants linked to phenotypes in animals and contributes to our understanding of phenotype-associated retroviral elements in domestic cats. The presence of the three DBE variants was reviewed in several feline breeding lines, thus assisting breeders with crucial information for testing their breeding stock and avoiding at-risk matings. Additional research is needed to identify the other still unknown feline DBE variants to help breeders and all stakeholders make informed decisions and establish guidelines regarding DBE cats. 

## Figures and Tables

**Table 1:** Main DBE breeding lines recorded in the cat species.

Lineage or Breed	Founder Cats	Genetic Background	White Spotting	Eye Phenotype	Hearing Phenotype in Heterozygous DBE Cats	Inheritance Characteristics	Homozygous DBE Cats	Sources and References
Altai	Fyodor (DSH)	Mixed	Minimal	Heterochromia or two blue eyes	Assumed to be normal (no BAER test available)	Autosomal dominant, incomplete penetrance, variable expressivity	Van-like * or white	Studied in this paper
Topaz	Roxi and Seymour (DSH)	Mixed	Minimal	Heterochromia or two blue eyes	Assumed to be normal (no BAER test available)	Autosomal dominant, incomplete penetrance, variable expressivity and pleiotropy	Bicolour **, van-like, or white; some of them are deaf; this group included homozygous and compound heterozygous cats	The line is probably lost (http://messybeast.com/DBE-topaz.htm, accessed on 11 April 2024) Not studied
Celestial	Roxi (DSH)	British shorthair/longhair, domestic shorthair/longhair	Minimal	Heterochromia or two blue eyes	Normal (BAER tested)	Autosomal dominant, incomplete penetrance, variable expressivity and pleiotropy	White; a single kitten was produced and died at birth	[12] Studied in this paper
British DBE (Seymour line)	Seymour (DSH)	British shorthair/longhair	Minimal	Heterochromia or two blue eyes	Assumed to be normal (no BAER test available)	Autosomal dominant, incomplete penetrance, variable expressivity and pleiotropy	Unknown, no homozygous cat was produced	Studied in this paper
British DBE (Nanotigr line)	Oliver (DSH) and his daughter named Amelia	British shorthair/longhair	Minimal	Heterochromia or two blue eyes	Assumed to be normal (no BAER test available)	Autosomal dominant, incomplete penetrance, variable expressivity	Van-like or white, some of them are deaf	Studied in this paper
British DBE (Igor line)	Igor Azur Dream (DSH)	British shorthair/longhair	Minimal	Heterochromia or two blue eyes	Deaf cats were reported (no BAER test available)	Autosomal dominant, incomplete penetrance, variable expressivity and pleiotropy	Unknown	Studied in this paper
British DBE (spontaneous variant, Nadeya line)	Nadeya Ermine Trace (BSH)	British shorthair/longhair	Minimal	Heterochromia or two blue eyes	Deaf cats were reported (no BAER test available)	Autosomal dominant, incomplete penetrance, variable expressivity and pleiotropy	Unknown	Studied in this paper
Persian DBE (Alaska line)	Seymour (DSH)	Persian	Minimal	Heterochromia or two blue eyes	Assumed to be normal (no BAER test available)	Autosomal dominant, incomplete penetrance, variable expressivity	Unknown, no homozygous cat was produced	Studied in this paper
Persian DBE (Cyrridwen line)	Marusya (DSH)	Persian and Exotic	Minimal	Heterochromia or two blue eyes	Assumed to be normal (no BAER test available)	Autosomal dominant, incomplete penetrance, variable expressivity	Unknown	Studied in this paper
Ragdoll DBE	Seymour (DSH)	Ragdoll	Minimal to bicolour ^$^	Heterochromia or two blue eyes	Assumed to be normal (no BAER test available)	Autosomal dominant, incomplete penetrance, variable expressivity	Unknown	Studied in this paper
Sphynx DBE	Unknown	Sphynx	Minimal	Heterochromia or two blue eyes	Assumed to be normal (no BAER test available)	Autosomal dominant, incomplete penetrance, variable expressivity	Unknown	Studied in this paper
Siberian DBE	Unknown	Siberian	Minimal to bicolour ^$^	Heterochromia or two blue eyes	Assumed to be normal (no BAER test available)	Autosomal dominant, incomplete penetrance, variable expressivity	Unknown	[12] Studied in this paper
Maine Coon DBE (Topaz line)	Roxi and Seymour (DSH)	Maine Coon	Minimal to bicolour ^$^	Heterochromia or two blue eyes	Assumed to be normal (no BAER test available)	Autosomal dominant, incomplete penetrance, variable expressivity	Unknown, no homozygous cat was reported ^#^	Studied in this paper
Maine Coon DBE (spontaneous variant, Dutch line)	Rociri Elvis (MCO)	Maine Coon	Minimal to bicolour ^$^	Heterochromia or two blue eyes	Deaf cats were reported (BAER tested)	Autosomal dominant, incomplete penetrance, variable expressivity and pleiotropy	Unknown, no homozygous cat was reported ^#^	[11] Studied in this paper
Maine Coon (Pillowtalk line)	Unknown	Maine Coon	Minimal to bicolour ^$^	Heterochromia or two blue eyes	Unknown	Autosomal dominant	Unknown	Studied in this paper
Maine Coon (Nahal line)	Nahal (domestic cat from Russia)	Maine Coon	No data available	No data available	No data available	No data available	No data available	Not studied

*: Van-like cats have a large amount of white in the fur, with the coloured areas being restricted to the back or the tail. In the classical van phenotype, the coloured areas are restricted to the tail and the head. **: Bicolour cats have a particular repartition of white and coloured areas in the fur, distinct from the well-known white spotting phenotype caused by the *w^S^* allele (http://messybeast.com/blue-eye-breeds.htm, accessed on 11 April 2024). ^#^: The mating of a dam from the Topaz line and a sire from the Dutch line produced a white kitten that died just after birth and showed an enlarged head and contracted and abnormal limbs. ^$^: In Siberian, Ragdoll, and Maine Coon cats, bicolour cats are born to a DBE parent with minimal white spotting and a non-DBE bicolour parent that is assumed to be white spotted due to the *w^S^* allele. An example is shown in Figure 1i. DSH: domestic shorthair, BSH: British shorthair, MCO: Maine Coon.

**Table 2:** Genotypes for the NC_018730.3:g.206975776_206975777insN[433] variant.

Phenotype	Lineage *	Breed	Founder Cats	Number	Genotype
DBE	Altai	Altai	Fyodor (outbred cat from Kazakhstan)	15	Heterozygous
White ^$^	Altai	Altai	Fyodor	2	Homozygous
DBE	Seymour	British (=6), Persian (=3, Alaska line), Ragdoll (=4)	Seymour (outbred cat from Russia)	13	Heterozygous
Latent	Seymour	Mixed breed (=1) and Persian (=1, Alaska line)	Seymour	2	Heterozygous
DBE	Nanotigr	British SH/LH	Unknown (Russia)	13	Heterozygous
White ^$^	Nanotigr	British SH/LH	Unknown (Russia)	1	Homozygous
Latent	Nanotigr	British SH/LH	Unknown (Russia)	3	Heterozygous
DBE	Unknown	Sphynx	Unknown	3	Heterozygous
DBE	Mixed (Seymour and Roxi)	Chinese Tank	Roxi and Seymour (outbred cats from Kazakhstan and Russia)	1	Heterozygous
White ^$^	Mixed (Seymour and Roxi)	Mixed breed	Roxi and Seymour	2	Heterozygous
DBE	Mixed (Seymour and Roxi)	Mixed breed	Roxi and Seymour	1	Heterozygous
DBE	Mixed (Seymour and Roxi)	Mixed breed	Roxi and Seymour	1	WT
DBE	Topaz (Seymour and Roxi)	Maine Coon	Roxi and Seymour	10	WT
DBE	Roxi	Celestial	Roxi (outbred cat from Kazakhstan)	20	WT
White ^$^	Roxi	Celestial	Roxi	1	WT
Latent	Roxi	Celestial	Roxi	1	WT
DBE	Dutch Maine Coon	Maine Coon	Purebred Maine Coon cat from the Netherlands	13	WT
DBE	Pillowtalk	Maine Coon	Unknown	2	WT
DBE	Unknown	Siberian	Unknown	4	WT
DBE	Igor	British SH/LH	Unknown (Russia)	6	WT
DBE	Nadeya	British SH/LH	Purebred British cat from Russia	2	WT
DBE	Cyrridwen	Exotic/Persian	Marusya (outbred cat from Russia)	1	WT
Total				117	
Control		British SH/LH		22	WT
Control		Celestial		2	WT
Control		Maine Coon		3	WT
Control		Siberian		2	WT
Control		Turkish Angora		1	WT
Control		Birman		1	WT
Control		Chartreux		2	WT
Control		Bengal		1	WT
Control		Devon Rex		1	WT
Control		Donskoy		1	WT
Control		Persian		2	WT
Control		Siamese		2	WT
Control		Sphynx		9	WT
Control		Domestic shorthair		2	WT
Control		Mixed breed		9	WT
Total				60	

DBE phenotype was defined by heterochromia or two blue eyes. Latent phenotype was defined by minimal white spotting and no blue eyes, but with a red eye effect during infancy. $ White or almost-white cat born to two DBE parents. WT: wild type, SH: shorthair, LH: longhair. * Lineage in accordance to http://messybeast.com/blue-eye-breeds.htm, accessed on 11 April 2024. Chinese Tank is a Munchkin–British SH/LH mixed breed.

## Data Availability

SNP genotyping data were deposited at OSF (https://osf.io/k798c/, accessed on 28 March 2024). Partial genomic sequence of *PAX3* intron 4 from a DBE cat (*Felis catus*) with the insertion was submitted to GenBank. The accession number is GenBank ID: PP332291. The whole-genome sequence of the DBE cat was submitted to SRA. The accession numbers are BioProject ID: PRJNA1073398 and BioSample ID: SAMN39921596.

## Supplementary Materials

The following supporting information can be downloaded at: https://www.mdpi.com/article/10.3390/ani14131845/s1, Figure S1: White and almost-white kittens born to two Altai DBE parents; Figure S2: Insertion of an RD-114 retrovirus element; Figure S3: Insertion of an RD-114 retrovirus LTR (long terminal repeat); Figure S4: Number of DBE cats genotyped for the three *PAX3* variants; Table S1: Twenty highest associated SNPs in the GWAS; Table S2: PCR and sequencing primers for *PAX3*; Table S3: Variants identified in *PAX3* coding region; Table S4: Structural variants identified in the candidate region; Table S5: DBE cats genotyped for two *PAX3* variants. Ref. [31] is cited in Figure S3.

## References

[1] Gandolfi B., Alhaddad H. (2015). Investigation of inherited diseases in cats: Genetic and genomic strategies over three decades. J. Feline Med. Surg..

[2] Lyons L.A. (2015). DNA mutations of the cat: The good, the bad and the ugly. J. Feline Med. Surg..

[3] David V.A., Menotti-Raymond M., Wallace A.C., Roelke M., Kehler J., Leighty R., Eizirik E., Hannah S.S., Nelson G., Schäffer A.A. (2014). Endogenous retrovirus insertion in the KIT oncogene determines white and white spotting in domestic cats. G3.

[4] Hou L., Pavan W.J. (2008). Transcriptional and signaling regulation in neural crest stem cell-derived melanocyte development: Do all roads lead to Mitf?. Cell Res..

[5] Baxter L.L., Watkins-Chow D.E., Pavan W.J., Loftus S.K. (2019). A curated gene list for expanding the horizons of pigmentation biology. Pigment. Cell Melanoma Res..

[6] Karlsson E.K., Baranowska I., Wade C.M., Salmon Hillbertz N.H., Zody M.C., Anderson N., Biagi T.M., Patterson N., Pielberg G.R., Kulbokas E.J. (2007). Efficient mapping of mendelian traits in dogs through genome-wide association. Nat. Genet..

[7] Yusnizar Y., Wilbe M., Herlino A.O., Sumantri C., Noor R.R., Boediono A., Andersson L., Andersson G. (2015). Microphthalmia-associated transcription factor mutations are associated with white-spotted coat color in swamp buffalo. Anim. Genet..

[8] Hofstetter S., Seefried F., Häfliger I.M., Jagannathan V., Leeb T., Drögemüller C. (2019). A non-coding regulatory variant in the 5′-region of the MITF gene is associated with white-spotted coat in Brown Swiss cattle. Anim. Genet..

[9] McFadden A., Vierra M., Martin K., Brooks S.A., Everts R.E., Lafayette C. (2024). Spotting the Pattern: A Review on White Coat Color in the Domestic Horse. Animals.

[10] Huang S., Song J., He C., Cai X., Yuan K., Mei L., Feng Y. (2022). Genetic insights, disease mechanisms, and biological therapeutics for Waardenburg syndrome. Gene Ther..

[11] Rudd Garces G., Farke D., Schmidt M.J., Letko A., Schirl K., Abitbol M., Leeb T., Lyons L.A., Lühken G. (2024). PAX3 haploinsufficiency in Maine Coon cats with dominant blue eyes and hearing loss resembling the human Waardenburg syndrome. G3.

[12] Abitbol M., Couronne A., Dufaure de Citres C., Gache V. (2024). A PAX3 insertion in the Celestial breed and certain feline breeding lines with dominant blue eyes. Animal Genet..

[13] Gandolfi B., Alhaddad H., Abdi M., Bach L.H., Creighton E.K., Davis B.W., Decker J.E., Dodman N.H., Ginns E.I., Grahn J.C. (2018). Applications and efficiencies of the first cat 63K DNA array. Sci. Rep..

[14] Purcell S., Neale B., Todd-Brown K., Thomas L., Ferreira M.A.R., Bender D., Maller J., Sklar P., de Bakker P.I., Daly M.J. (2007). PLINK: A tool set for whole-genome association and population-based linkage analyses. Am. J. Hum. Genet..

[15] McKenna A., Hanna M., Banks E., Sivachenko A., Cibulskis K., Kernytsky A., Garimella K., Altshuler D., Gabriel S., Daly M. (2010). The Genome Analysis Toolkit: A MapReduce framework for analyzing next-generation DNA sequencing data. Genome Res..

[16] Chen X., Schulz-Trieglaff O., Shaw R., Barnes B., Schlesinger F., Källberg M., Cox A.J., Kruglyak S., Saunders C.T. (2016). Manta: Rapid detection of structural variants and indels for germline and cancer sequencing applications. Bioinformatics.

[17] Untergasser A., Cutcutache I., Koressaar T., Ye J., Faircloth B.C., Remm M., Rozen S.G. (2012). Primer3—New capabilities and interfaces. Nucleic Acids Res..

[18] Corpet F. (1988). Multiple sequence alignment with hierarchical clustering. Nucleic Acids Res..

[19] Moore S., Ribes V., Terriente J., Wilkinson D., Relaix F., Briscoe J. (2013). Distinct regulatory mechanisms act to establish and maintain PAX3 expression in the developing neural tube. PLoS Genet..

[20] Jern P., Coffin J.M. (2008). Effects of retroviruses on host genome function. Annu. Rev. Genet..

[21] Degenhardt K.R., Milewski R.C., Padmanabhan A., Miller M., Singh M.K., Lang D., Engleka K.A., Wu M., Li J., Zhou D. (2010). Distinct enhancers at the Pax3 locus can function redundantly to regulate neural tube and neural crest expressions. Dev. Biol..

[22] Pontius J.U., Mullikin J.C., Smith D.R., Lindblad-Toh K., Gnerre S., Clamp M., Chang J., Stephens R., Neelam B., Agencourt Sequencing Team (2007). Initial sequence and comparative analysis of the cat genome. Genome Res..

[23] Medstrand P., van de Lagemaat L.N., Mager D. (2002). Retroelement distributions in the human genome: Variations associated with age and proximity to genes. Genome Res..

[24] Cui Y.Z., Man X.Y. (2023). Biology of melanocytes in mammals. Front. Cell Dev. Biol..

[25] Hauswirth R., Haase B., Blatter M., Brooks S.A., Burger D., Drögemüller C., Gerber V., Henke D., Janda J., Jude R. (2012). Mutations in MITF and PAX3 cause “splashed white” and other white spotting phenotypes in horses. PLoS Genet..

[26] Hauswirth R., Jude R., Haase B., Bellone R.R., Archer S., Holl H., Brooks S.A., Tozaki T., Penedo M.C., Rieder S. (2013). Novel variants in the KIT and PAX3 genes in horses with white-spotted coat colour phenotypes. Anim. Genet..

[27] Epstein D.J., Vogan K.J., Trasler D.G., Gros P. (1993). A mutation within intron 3 of the Pax-3 gene produces aberrantly spliced mRNA transcripts in the splotch (Sp) mouse mutant. Proc. Natl. Acad. Sci. USA.

[28] Zlotogora J., Lerer I., Bar-David S., Ergaz Z., Abeliovich D. (1995). Homozygosity for Waardenburg syndrome. Am. J. Hum. Genet..

[29] Wollnik B., Tukel T., Uyguner O., Ghanbari A., Kayserili H., Emiroglu M., Yuksel-Apak M. (2003). Homozygous and heterozygous inheritance of PAX3 mutations causes different types of Waardenburg syndrome. Am. J. Med. Genet. A.

[30] Salah S., Meiner V., Abumayaleh A., Asafra A., Al-Sharif T., Al-Fallah O., Hasasneh B., Zlotogora J. (2022). Biallelic variants in PAX3 cause Klein syndrome. Clin. Genet..

[31] Yoshikawa R., Sato E., Igarashi T., Miyazawa T. (2010). Characterization of RD-114 virus isolated from a commercial canine vaccine manufactured using CRFK cells. J. Clin. Microbiol..

