# Supplementary figures and images for: Different Founding Effects Underlie Dominant Blue Eyes (DBE) in the Domestic Cat
Source: Animals (Basel). 2024 Jun 21;14(13):1845. doi: 10.3390/ani14131845 (PMC11240321; doi:10.3390/ani14131845)

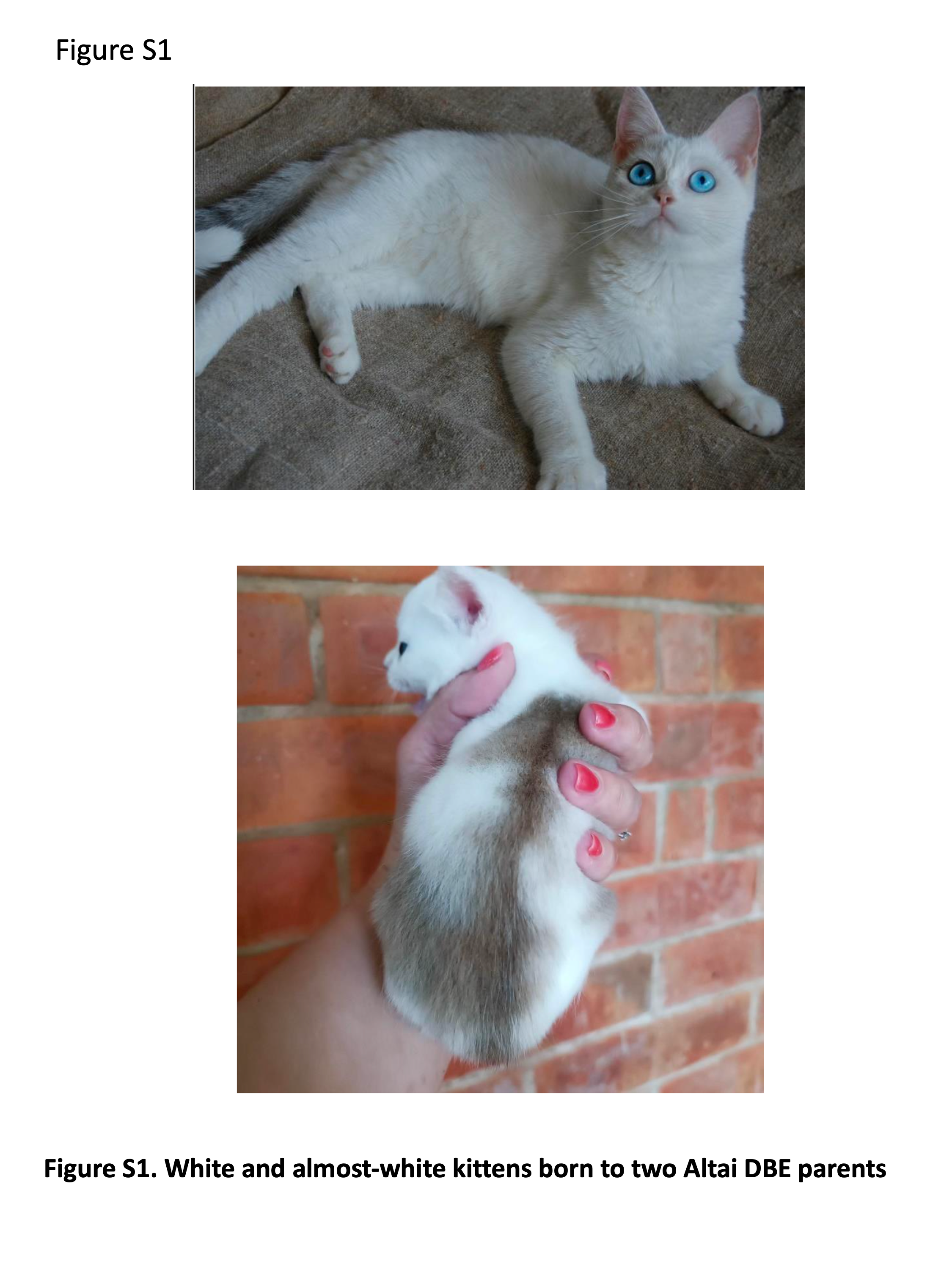

Supplement: Supplementary file 1 [file animals-14-01845-s001.zip › Figure_S1.tiff]

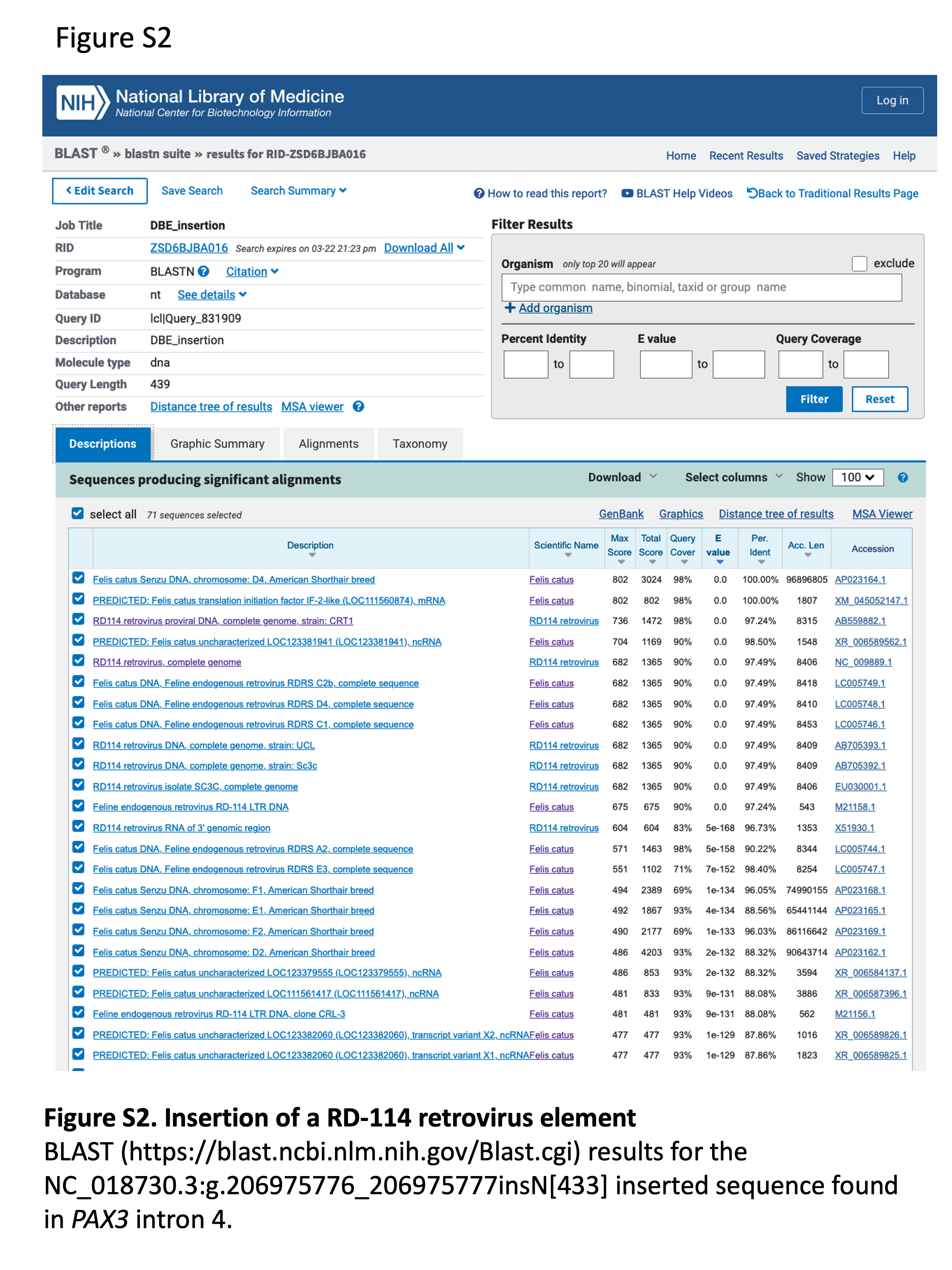

Supplement: Supplementary file 1 [file animals-14-01845-s001.zip › Figure_S2.tiff]

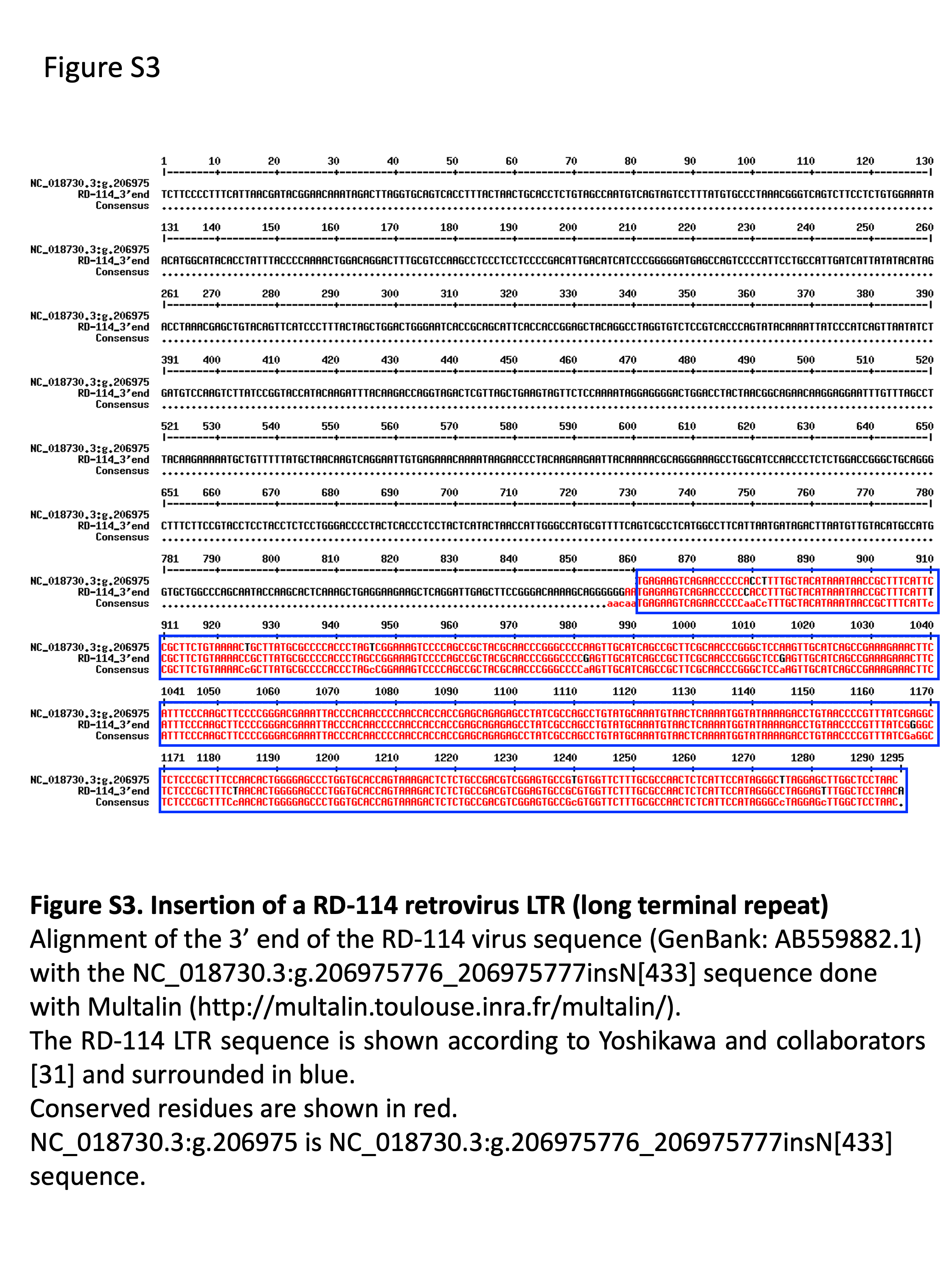

Supplement: Supplementary file 1 [file animals-14-01845-s001.zip › Figure_S3.tiff]

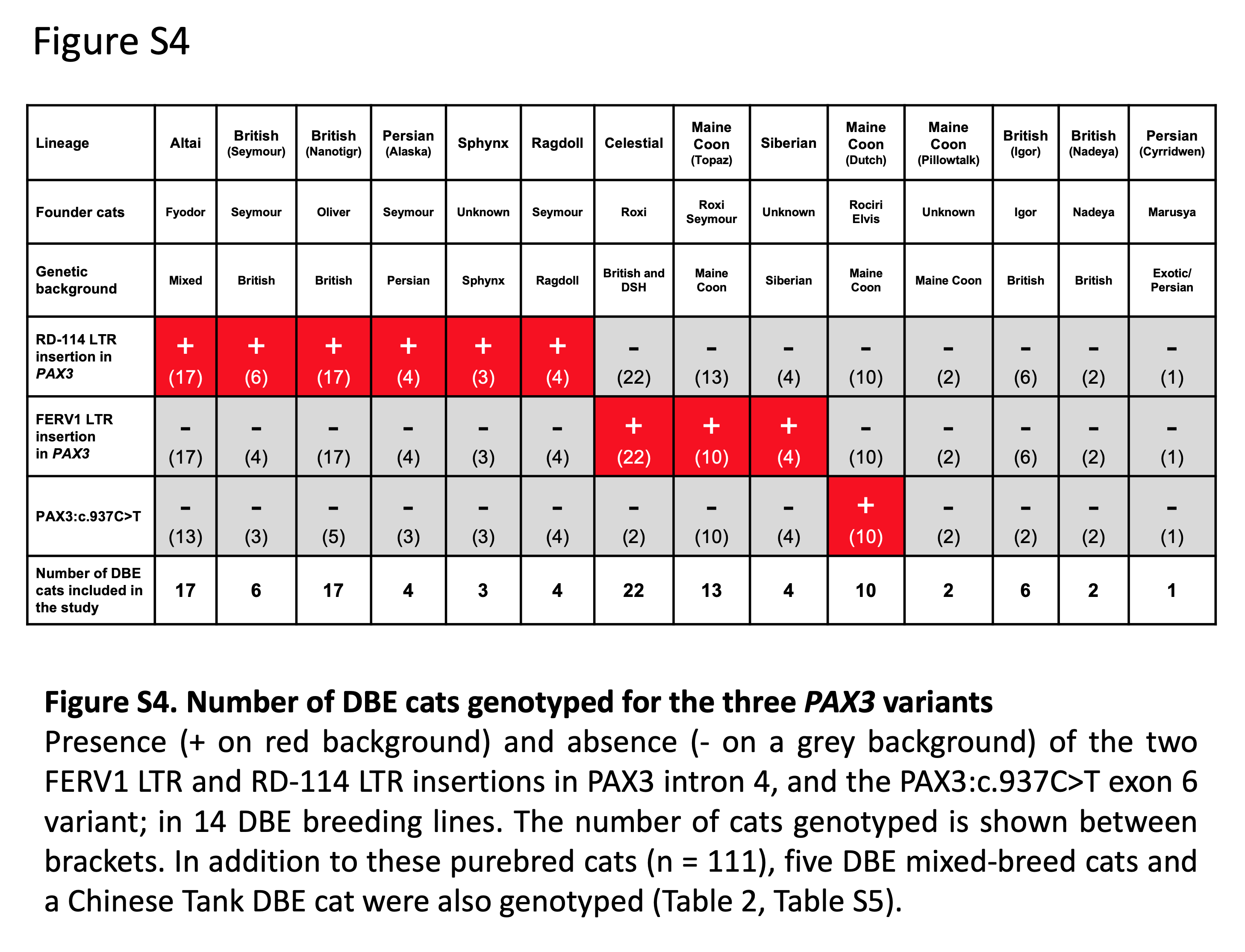

Supplement: Supplementary file 1 [file animals-14-01845-s001.zip › Figure_S4.tiff]
